# Experiences and Reported Outcomes of Patients and Caregivers Enrolled in an Integrated Care Program for Thoracic Surgery: A Qualitative Evaluation

**DOI:** 10.5334/ijic.6540

**Published:** 2023-05-04

**Authors:** Meghan O’Neill, Kathy Kornas, Catherine Liang, Lori Diemert, Tsoleen Ayanian, Melissa Chang, Laura C. Rosella

**Affiliations:** 1 Dalla Lana School of Public Health, University of Toronto, Toronto, Ontario, CA; 2 University Health Network, Toronto, Ontario, CA; 3 ICES, Toronto, Ontario, CA; 4 Institute for Better Health, Trillium Health Partners, Mississauga, Ontario, CA; 5 Laboratory Medicine and Pathobiology, Temerty Faculty of Medicine, University of Toronto, CA

**Keywords:** integrated care, person-centered care, thoracic surgery, qualitative study, evaluation

## Abstract

**Background:**

Health care delivery is often poorly coordinated and fragmented. Integrated care (IC) programs represent one solution to improving continuity of care. The aim of this study was to understand experiences and reported outcomes of patients and caregivers in an IC Program that coordinates hospital and home care for thoracic surgery.

**Methods:**

A process evaluation was undertaken using qualitative methods. We conducted semi-structured interviews with 10 patients and 8 caregivers who received IC for thoracic surgery and were discharged between June 2019 and April 2020. A phenomenological approach was used to understand and characterize patient and caregiver experiences. Thematic analysis began with a deductive approach complemented by an inductive approach.

**Results:**

Four major themes evolved from patient and caregiver interviews, including 1) coordination and timeliness of patient care facilitated by an IC lead; 2) the provision of person-centred care and relational continuity fostered feelings of partnership with patients and caregivers; 3) clear communication and one shared digital record increased informational continuity; and 4) impacts of IC on patient and caregiver outcomes.

**Conclusions:**

Patients and caregivers generally reported this IC Program met their health care needs, which may help inform how future IC programs are designed.

## Introduction

Existing models of care delivery are often inadequately designed to meet the diverse range of health and social supports required for individuals with complex needs. Limitations include care fragmentation, poor coordination, and lack of patient and caregiver involvement [1]. This is problematic for individuals with complex needs, such as those undergoing thoracic surgery, who require care from different providers in various health care settings over extended periods of time [2]. In Ontario, and much of Canada, home and community care organizations are siloed, operating separately from hospitals, creating multiple points of exchange for patients and caregivers [2,3,4]. The current model of care delivery is reported to be rigid, with limited flexibility to scale up resources based on unique patient needs [4]. Additionally, informational exchange between primary care and the hospital is impeded by differences in information technology [5]. Collectively, these limitations have been linked to negative outcomes, including increased emergency department (ED) visits, higher risk of mortality, and unfavourable care experiences [6,7,8,9].

Health systems internationally have committed to implementing more sustainable models of care to improve patient experiences and health outcomes [10]. Integrated Care (IC) models may represent a promising solution, with the aim of creating a system of coordinated and continuous care across providers and facilities, tailored to the unique needs of patients and caregivers [11]. A central component to IC models includes person-centered care as a critical component [12]. Therefore, the person-centered lens of IC, conceptualized by the United Kingdom National Health Services best emulates the aims of the thoracic surgery IC Program described in this study [13].

Reviews on patient and caregiver experiences in the health care system broadly have found several challenges with information exchange between providers and care settings, confusion with roles and responsibilities of providers and caregivers, and disorganized discharge planning [14]. A recent systematic review on patient experiences with care integration in the United Kingdom highlighted the need for future research that provides context to the patient experience, details on the integration processes, and the use of validated patient experience measures [15]. Designing IC programs with attention to the patient-caregiver dyad is important for reducing caregiver burden and improving overall caregiver engagement and satisfaction. The majority of home care is provided by informal caregivers; thus supporting caregivers through person-centred care plans has been identified as a key component in sustaining community-based care [16]. Perceptions of caregivers and their wellbeing have been extensively studied but understanding caregivers’ experiences in the context of IC has not been well-characterized. To evaluate whether IC models meet the needs of patients and caregivers requires a combination of quantitative and qualitative research methods [17]. Internationally, there have been few studies aimed at evaluating IC programs for thoracic surgery, the majority of which used quantitative methods [18,19]. The objective of this study was to evaluate patients and caregivers’ experiences and reported outcomes in the thoracic surgery IC Program, which aims to integrate in-hospital care with proactive discharge planning to improve continuity of care in the community setting.

## Methods

### Research methodology

We undertook a qualitative process evaluation to assess whether the IC Program is being implemented and delivered as intended and the mechanisms by which the Program may impact different outcomes [20]. This approach is well positioned to enhance our understanding of patient and caregiver experiences and to inform program improvements. An evaluation matrix was used to inform data collection and analysis. An evaluation matrix is a planning tool that connects each evaluation question to the sources of data for answering that question [21]. A qualitative phenomenological approach [22] was used in this study since this approach centres the participants subjective lived experiences and seeks to explore the meanings of those experiences. This study is embedded within a larger mixed-methods evaluation; further details about the IC program and health care provider experiences are described elsewhere [23].

### Positionality and reflexivity

The evaluation team included graduate students, researchers, and health care administrators, with roles, preconceptions, and viewpoints that may have impacted the data analysis and conclusions. Evaluation team members are trained in qualitative data analysis using reflexive note taking as one means to track positionality. To further enhance the reflexivity and reliability of our approach, we held regular working group meetings with broader Program stakeholders (e.g., program staff, directors, and patient representatives) to discuss and solicit feedback on emerging themes.

### Program description

In June 2019, the University Health Network, a large University-affiliated hospital in Ontario, Canada, implemented an IC Program in the thoracic surgery department. The program was modelled after an Integrated Comprehensive Care program at St. Joseph’s Healthcare in Hamilton, Ontario and was subsequently modified through collaboration with patients and stakeholders across care sectors [19]. The program has four defining features: 1) an IC lead who is a registered nurse and central coordinator in the care model that helps patients navigate their care journey from the hospital to the community; 2) a 24/7 phone line available to patients and caregivers to answer questions; 3) one shared digital record; and 4) an integrated funding package containing bundled services and payment. Depending on individual circumstances and the type of surgical procedure, patients are enrolled into low, medium, or high IC care paths. Each care path is distinguished by the levels of support needed and resources offered, with greater supports available to patients in the high path, compared to the low path. Services provided in the IC Program include support in the community setting with check-in calls from the IC lead, and depending on the care path, patients may also receive additional pre-rehabilitation services, including home care visits from a nurse, dietician, physiotherapist and/or personal support worker (see Table 1).

**Table 1:** Description of the criteria for enrollment into low, medium, and high care path with corresponding resources and supports provided to patients.


THORACIC SURGERY PATH	INTERVENTION	RESOURCES AND SUPPORTS

**Low**	Lung & Mediastinal Resection (VATS/RATS) Thymectomy (Video Assisted) Pleuroscopy Other miscellaneous low volume surgeries	Patient is on pathway for 30 days (but can be extended for up to 90 days) Proactive IC lead check-in calls + additional calls and homecare supports as the need arises

**Medium**	Decortication (Video Assisted) Pneumonectomy Thoracotomy Sternotomy Tracheal Resection & Repair (includes t-tube) Lung Volume Reduction Trans Thoracic Hiatus Hernia Repair; Fundoplication; Peroral Endoscopic Myotomy; Esophageal Repair Chest Reconstruction (with or without flap)	Patient is on pathway for 60 days (but can be extended for up to 90 days) Proactive check-in calls Standard homecare supports (nursing, allied health, personal support, supplies and equipments) + additional calls and homecare supports as the need arises

**High**	Extrapleural Pneumonectomy Pulmonary Endarterectomy Esophagectomy	Patient is on pathway for 90 days Proactive check-in calls Standard homecare supports (nursing, allied health, personal support, supplies and equipments) Pre-hab/pre-surgery homecare, as needed + additional calls and homecare supports as the need arises


### Development of interview guide

Two separate interview guides were developed for patients and caregivers, with questions and probes used to capture information on indicators identified in the evaluation matrix which was informed by the evaluation working group and the World Health Organization’s Framework on Integrated People-Centred Health Services [24] (S1 Table). Interview questions were leveraged from existing standardized tools [25,26,27,28,29,30,31]; however, question wording was adapted where necessary to allow for open-ended responses.

### Participant recruitment and interviews

Two IC leads from the hospital’s thoracic surgery department facilitated the recruitment of a sample of patients and caregivers. To include a diverse study sample that reflected a broad range of experiences, maximum variation sampling was used, which also ensured that our sample included both patients and caregivers from different IC paths (i.e., low, medium, and high) [32]. Patients and caregivers who finished their care path and were discharged between June 30, 2019, and April 30, 2020, were eligible. The final sample size was 10 patients (4 low path, 4 medium path, 2 high path) and 8 caregivers (3 low path, 3 medium path, and 2 high path). The sample included 4 (22.2%) males and 14 (77.8%) females. Two experienced researchers from the external evaluation team completed the interviews by telephone between April 9, 2020, and June 5, 2020. All participants received a gift card, valued at $20 Canadian dollars. Median patient interview length was 26 (IQR:19–35) minutes and median caregiver interview length was 39 (IQR:37–44) minutes.

### Qualitative analysis

All interviews were audio-recorded and transcribed verbatim. Information from patient and caregiver interviewers were qualitatively analyzed using a data reduction, data display, and conclusion drawing approach [33]. The first step in the data reduction process involved selecting, focusing, and abstracting relevant pieces of information from the transcripts into more manageable groups of similar information. This was achieved through the process of thematic analysis, which was used to identify, categorize, and describe themes. First, we used a deductive approach to explore the transcripts for themes related to indicators defined in the evaluation matrix, in combination with an inductive approach to identify themes that emerged from the data. A coding manual [34] was developed to abstract and synthesize the data using a preliminary list of codes constructed to reflect the indicators in the evaluation matrix (S2 Table). Two authors, independently coded one patient and one caregiver transcript and met to review intercoder agreement and refine the coding guide. Further rounds of double coding were completed until there was very strong agreement to proceed with independent coding of the remaining transcripts [35].

The second step involved the creation of a data display which is a table that displays the data in an organized and compressed manner, promoting relationships and patterns within the data. Finally, conclusion drawing involved an overall assessment of the analysed data to identify what learnings can be drawn from it. As the data display and conclusion drawing progressed, preliminary findings were brought to the evaluation working group to solicit feedback. NVivo 12 Software was used to facilitate qualitative data analysis. This study is reported in line with the Consolidated Criteria for Reporting Qualitative Research (COREQ) checklist [36,37].

### Patient Involvement

This evaluation was undertaken with input from an evaluation working group comprised of patient representatives, researchers and practitioners. The evaluation team met with the working group throughout the evaluation process to finalize the evaluation questions, advise on the methods and indicators, as well as guide the interpretation of findings and formulation of recommendations for the IC Program.

### Ethics approval

This evaluation was deemed a quality improvement project as described in the Tri-Council

Policy Statement V.2; therefore, the project received a waiver from the University Health Network (UHN) and University of Toronto Research Ethics Boards.

## Results

**Figure 1:**
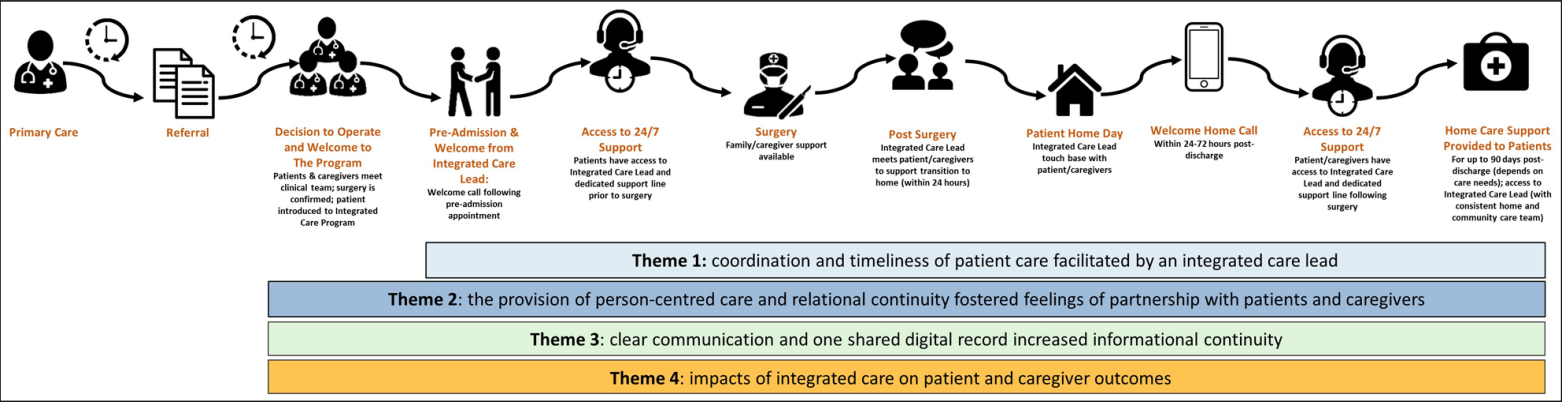
Themes mapped across the patient trajectory.

The responses from patients and caregivers were grouped into four major themes, including 1) coordination and timeliness of patient care facilitated by an IC lead; 2) the provision of person-centred care fostered feelings of partnership with patients and caregivers; 3) clear communication and one shared digital record increased informational continuity; and 4) impacts of IC on patient and caregiver reported outcomes. A conceptual diagram, depicting the care journey with corresponding themes is displayed in Figure 1.

### Theme 1: Coordination and timeliness of patient care facilitated by an integrated care lead

Most patients and caregivers in our sample felt they received well-coordinated and timely care. Coordination of care was facilitated by having the IC leads function as the link between the community and hospital. Patients and caregivers expressed that they felt they could call the IC lead and receive a timely response to their questions. For example, one patient recalled contacting the IC lead because they were having reoccurring episodes of nausea and vomiting. The IC lead promptly arranged for the patient to return to the hospital the following day, where a minor surgery was needed to address the issue.

In our interviews with patients and caregivers, we observed evidence that the IC leads played a vital role in supporting several aspects of care coordination, including assessing changes in patient health status, responding to concerns from patients and caregivers, and coordinating follow-up care. For example, a patient recalled contacting the IC lead about a blockage in a tube and the IC lead arranged for a nurse to arrive at their home a few hours later to correct the problem. In some cases, patients said they called the IC lead to communicate new symptoms that may be a cause for concern. For example, one patient concerned about their incision sent pictures of it to the IC lead, and a follow-up appointment was arranged with their surgeon:

*“I was running into a problem and there was a bulge by one of my incisions, and [the IC lead] looked at the pictures of it and showed it to my thoracic surgeon, who in turn called me and then his secretary had me come in a week later to have a look at it. So, that was good, and I think I needed her pull to get that to happen because I had called the surgeon’s office and had gotten nowhere myself. I left a message. I didn’t get any response.”* (Patient, medium care path)

Several patients and caregivers noted that the check-in calls from the IC lead were helpful because they allowed them to voice concerns and receive timely follow-up care. The 24/7 support line was typically utilized by patients, although a few caregivers also accessed this resource and reinforced reflections from patients about effective and proactive coordination of care. For example, one caregiver contacted the IC lead after noticing a prescription was missing once the patient was discharged, and said the IC lead immediately arranged for the prescription to be sent to the pharmacy:

*“The day we were discharged there was one error. One of the prescriptions was not included and I noticed this when we got home. So I called the office, I spoke with [the IC lead] and she worked very hard over the next two hours to write emails to the doctor, and the doctor actually sent the script to the pharmacy and it was all looked after. It was all resolved, but I wouldn’t have got it resolved if I didn’t have the 24/7 support number that I was given.”* (Caregiver, medium care path)

### Theme 2: The provision of person-centred care and relational continuity fostered feelings of partnership with patients and caregivers

Most patients and caregivers reported sufficient levels of support and information to manage their care post-discharge. However, a patient who was introduced to the Program post-surgery said they did not have enough information in advance to prepare for their discharge, particularly in regard to their dietary needs: *“I think [pre-op] is when [program staff] should have given me the timeline about foods, how long I had to be X number of weeks doing straight liquids, X number of weeks doing moist food and ideas of what I could eat or consume. I would have been better prepared. So, when I came home, we were scrambling, and I was in no shape to be scrambling.”* (Patient, medium care path). Overall, the majority of caregivers said they felt involved as much as they wanted to be in decisions about the patients care. Patients also generally agreed that their caregivers and families were appropriately involved in decisions about their care and felt that their preferences were respected. Some patients and caregivers shared examples of how doctors and other hospital staff listened and were attentive to the patients’ preferences. For example, a patient shared how the clinical team listened and worked with them to address pain management: *“Sometimes the pain was just unbelievable, and I would talk to the doctors and say, ‘Can we try this? Can we do this?’ And they were all very receptive. And I would say, ‘This stuff is making me nauseated; it’s not working well’. And they would say, ‘well let’s try this and let us know how it works out’. I was included [in decisions] and what I had to say was important to them.”* (Patient, high care path). One caregiver shared that they felt that the IC Program partnered with them in caring for the patient:

*“I kept after [the patient] and she didn’t really appreciate it at times but I knew that she wasn’t meeting the calorie intake so I would pick up something like Ensure or Boost in the plus format that had extra calories… She didn’t want to take that and then [I] felt very much vindicated when the Dietician would call every second week and she’d describe what she was eating and [the Dietician] would say ‘nope, that’s not enough, you should be taking a supplement’. So, you know, it was all good to have resources to back you up…it took a load off me because it made it easier for me to come back home with another pack of Ensure from the pharmacy.”* (Caregiver, medium care path)

Patients and caregivers were followed by the IC leads throughout their entire care trajectory, from pre-hospitalization to post-discharge. This relational continuity helped to build and foster rapport with patients and caregivers. By proactively addressing concerns in real-time, a few patients recounted that the IC lead may have helped to avoid an unnecessary trip to the ED: *“If I had some kind of a problem, I would call the [IC lead] to help me with my issue and avoid making a trip to the ED, you know”* (Patient, medium care path). In most situations, the IC lead was able to re-assure the patient that their symptoms did not necessitate a visit to the ED. In some cases, the IC leads were able to move up an appointment with their surgeon to accommodate changes in the patient’s health status. Caregivers expressed immense gratitude for the IC lead, who was a bridge between the hospital and community setting. The importance of rapidly arranging for the patient to return to the hospital that completed the thoracic surgery, ensured that providers were up to date about the patient’s health and treatment plan:

*“There’s a program with us regarding my mom’s issue and they know everything about my mom, and I don’t need to tell [other providers] the story; start from the very beginning. It’s really amazing because if something were to happen to my mom, I could contact her family doctor, but I would still need to make an appointment or go to the ED, and those aren’t the best choices because I would need to let them know what happened; start from the very beginning. But [the IC lead] knows everything.”* (Caregiver, low care path)

### Theme 3: Clear communication and one shared digital record increased informational continuity

During their hospital stay, patients and caregivers said they experienced good communications about their care between doctors, nurses, and other hospital staff. Patients and caregivers generally felt that doctors and other staff answered their questions, provided enough information, and explained things in a way they could understand. For example, one caregiver reflected: *“You know, we had no problems with any of the nurses. If you asked them a question, they always answered, not yes or no, it was always kind of detailed. They would explain everything to you and why this is happening and so [the communication] was good.”* (Caregiver, high care path). Patients and caregivers appreciated having their questions answered by the IC lead when they were discharged from the hospital, and frequently expressed appreciation in the staff’s knowledge of their medical history so that they did not have to re-explain things to different providers:

*“I didn’t have to go to my family doctor and explain the situation to my family doctor. That would have been a nightmare because number one you’ve just had an hour surgery, you’ve been in there for ten days, you need transportation to get there, you’re not well. And the family doctor probably wouldn’t even touch you because it was the thoracic surgeon who did all that so they most probably wouldn’t have done anything and told me to go see the thoracic surgeon on Monday, you know what I mean? This is so much easier. I phoned the IC lead, gave them symptoms, and they took care of it.”* (Patient, high care path)

When asked about their experiences with communication, most patients felt that health care providers worked as a team:

*“If something wasn’t right, they discussed it with me and tried to improve it. It seemed they all worked together as a team, and it went so smoothly. They were all on the same page. Everybody knew what was going on, what the treatment was, what the end result was…there wasn’t one person saying oh, you need this. No, they were all together. All on one page. All saying the same thing, so it was reinforced all the time.”* (Patient, high care path).

Patients and caregivers generally felt that health care providers within the hospital were informed and up to date about their care, and many felt this was facilitated by having a shared electronic patient record. For example, a caregiver said that their surgeon was able to access the patients medical record with another physician in the hospital to review a concern they brought up about medication dosage. Another patient elaborated on the electronic patient portal containing their medical records:

*“I know in the [hospital] portal I had all the information as well; so everything that they put into their notes I would see and my other physicians at [the hospital I had surgery at] could see whatever I had at [partnering hospital], so they were well informed when I had to have meetings with them about what was going on and I know [my thoracic surgeon] and [my other doctor] communicated a couple of times, so as far as I know communication was going really well.”* (Patient, low care path)

### Theme 4: Impacts of integrated care on patient and caregiver outcomes

Most patients in our sample felt supported and confident in their ability to self-manage their health. Patients frequently reflected on the utility of the IC leads in increasing patient confidence to self-manage their health, particularly during the post-discharge period. Patients reported feelings of *“comfort”, “support”, “security”*, and *“reassurance”* when reflecting on the availability and timeliness of the IC lead’s responses to their questions. Even if patients did not contact the IC lead, which was more common among low path patients, they noted it was comforting to have this resource available:

*“I can’t overemphasize that it was a very great comfort to know that you had a link to somebody that can answer questions if you have them. Even if you don’t have [questions] it’s nice to know, it’s like a lifeline that’s there if you need it but hopefully you don’t need it.”* (Patient, low care path)

In addition to the IC lead, patients in the high care path commented on the importance of having a dietician and home care team, stating that their support was influential in their ability to self-manage their health and accelerate their physical recovery post-surgery:

*“If I had a problem, I’d call the nutritionist and they would fix it. I wasn’t left on my own to try and figure things out…the biggest problem is learning how to eat again, and she helped me a lot. I couldn’t have done it without her. I got better faster.”* (Patient, high care path).

While most caregivers felt prepared for their role to support the patient during the post-discharge period, a few expressed that they felt the program was primarily for the patient and suggested it would have been helpful to know that these resources were also available to them to utilize and to empower them as caregivers:

*“I didn’t consider myself part of the integrated care program. It was more like okay, so [the patient] is going to be a part of the program and I’m kind of just like an onlooker. I think another thing that would be useful is making [caregivers] know that they’re a part of this as well and kind of also empowering them to be an active part of this because I know from my mom and I, we would feel like passive caregivers and a lot of the times we would just ask [the patient] to either call them or ask them questions and it really depended on [the patient] doing it. [The patient’s] health depended on them taking action or not, and a lot of times they wouldn’t take action on those things, and then we wouldn’t do anything about it. We would just kind of sit back and just say okay, that’s it, but I think if we were more active caregivers, I think maybe I would have taken the initiative to call the IC leads or somebody in that department and ask them what we could do.”* (Caregiver, medium care path).

About half of the caregivers in our sample felt they successfully navigated their role and that caring for the patient was not too burdensome or stressful. Some caregivers mentioned that they had previous caregiving experience and that they were familiar with hospital processes and advocating for the patient, which they felt built resiliency and contributed positively to their emotional wellbeing. About half of caregivers felt that their mental and physical health was impacted by caring for the patient. One caregiver stated that their health was negatively impacted because they changed their own eating habits to coincide with the patient’s new diet and were frequently worried about the patient’s health. Two other caregivers commented on the stresses of watching a loved one go through treatment and that caring for the patient may have impacted their own mental health:

*“I think in general my mental health was impacted the most. My physical health was fine, but I think mentally speaking I was quite drained and extremely stressed out, the usual [stress] I guess that comes with caregiving.”* (Caregiver, medium care path).

The general sense among caregivers was that they had to manage or *‘deal with’* the situation the best they could. There was reluctance to ask for support and some felt their physical or emotional needs were less important than the patient’s needs: *“There was no support or anything for me. I just dealt with it. I never mentioned myself.”* (Caregiver, high care path). Multiple caregivers indicated that they felt the IC Program was more patient-focused and they were unaware of resources available to support caregiver wellbeing.

## Discussion

We observed that from the perspective of patients and caregivers, the thoracic surgery IC Program achieved objectives of providing well coordinated and continuous care, specifically during the post-discharge period. Patients generally felt prepared to be discharged from the hospital, felt confident to manage their care at home, and felt comforted by having access to an IC lead. The IC leads played an important role in the provision of person-centred care, relational continuity, and ensuring informational continuity across care settings. However, some caregivers felt their physical and emotional health was impacted by caregiving and they were unaware of resources available to support their wellbeing.

A systematic review on patient experiences in IC found that poor care experiences occur when there is limited flexibility to meet individual patient needs or when the patient is expected to conform or adhere to a particular model of care [15]. In this IC program, we observed positive perceptions amongst patients and caregivers when reflecting on the supports and resources offered, highlighting the importance of designing care plans that are responsive to patient and caregiver needs. Our findings are consistent with other Canadian evaluations of IC initiatives, that found patients and caregivers perceived improvements in their emotional wellbeing with access to a 24/7 phone line [38]. We observed that some caregivers felt their needs were less important than the patients which is consistent with other literature that suggests caregivers may be reluctant to voice their own needs [39]. While caregiving can be rewarding, existing qualitative studies have demonstrated that caregivers’ health and wellbeing can be negatively impacted, highlighting the necessity of tailored community supports and resources [40]. Such supports may include ensuring caregivers know they are able to use the 24/7 support line for questions, access to peer support, and e-learning resources [41]. Many evaluations show that caregivers feel unengaged and underprepared during the discharge process [42,43]. Despite this, caregivers in the IC program reported that they felt they were provided with sufficient levels of education and that they were meaningfully involved in decisions.

With increased reliance on caregivers in models of IC, it is important to strengthen caregiver engagement and ease of access to the patients care team post-discharge, which has resulted in improvements to patient safety and health outcomes [44,45,46]. This barrier in access to the patients care team was addressed in the thoracic surgery IC Program through the provision of a 24/7 phone line and regular calls from the IC lead. Our findings corroborate existing evaluations of thoracic surgery IC programs that highlight how the care coordinator was able to provide instructions on chest tube care, pain management, and how managing follow-up care helped to avoid unnecessary use of the ED [47,48]. Several systematic reviews have demonstrated that hospital readmissions decline when the patient is engaged [49], empowered [50,51], and leverages the patient and caregiver capacity for community care [50,52]. We observed strong and positive relationships between patients and the IC leads, which was facilitated through sustained communications with the patient throughout their entire care journey.

This study fills an important gap identified by a recent systematic review [15] on patient experiences within the context of IC. Specific strengths include the use of qualitative methods to provide rich contextual descriptions of patients’ and caregivers’ experiences with IC. These experiences were measured using validated tools that provided detailed insight into the impact of the thoracic surgery IC Program on patients and caregivers, as well as challenges and opportunities to improve the delivery of care. The IC evaluation was informed by a diverse working group, composed of researchers, providers, and patient partners with an iterative feedback process throughout the design of the evaluation. This study should be interpreted considering certain limitations. Although interviews with patients and caregivers provided rich information, it is possible that the views are not reflective of all patients and caregivers enrolled in the IC Program given the relatively modest sample. In consideration of this limitation, we interviewed participants until we reached data saturation, whereby new information echoes what was expressed in previous interviews [53]. Additionally, this IC program was implemented in the context of Canadas publicly funded health care system, therefore findings may not be directly translatable to other health systems.

## Conclusions

The evaluation of the IC Program showed that from the perspective of patients and caregivers, the thoracic surgery IC model supported the delivery of comprehensive, continuous, and person-centred care. Reflections from caregivers suggest the need to ensure that caregivers feel empowered and supported. As a result of the promising findings from this qualitative evaluation (and the broader mixed methods study), the IC Program will expand to other patient groups at the University Health Network, including other surgical specialities.

## Additional Files

The additional files for this article can be found as follows:

10.5334/ijic.6540.s1 S1 Table. Patient and caregiver interview guide and corresponding evaluation indicators.

10.5334/ijic.6540.s2 S2 Table. Qualitative analysis coding manual.
