# Supplementary material for: Experiences and Reported Outcomes of Patients and Caregivers Enrolled in an Integrated Care Program for Thoracic Surgery: A Qualitative Evaluation
Source: Int J Integr Care. 2023 May 4;23(2):11. doi: 10.5334/ijic.6540 (PMC10162348; doi:10.5334/ijic.6540)
Supplement: S1 Table. — Patient and caregiver interview guide and corresponding evaluation indicators. [file ijic-23-2-6540-s1.pdf]

S1 Table. Patient and caregiver interview guide and corresponding evaluation indicators

Indicator	Interview Question	
	**Patients**	**Caregivers**
	**Experience pre-hospital admission**	
Experiences of the Integrated Care program: Pre-hospital admission process<br><br>Perceived efficacy: Interactions and communications<br><br>Perceived efficacy: Education & knowledge<br><br>Outcome: Perceived patient preparedness	**Question 1a:** Can you tell me why you decided to participate in the Integrated Care program?<br><br>**Question 1b:** Before coming to the hospital, did you have enough information about what was going to happen in the Integrated Care program?<br><br>*Probe: Did you have enough information about what was going to happen during the admission process?*	**Question 1.** Before coming to the hospital, did you have enough information about what was going to happen in the Integrated Care program?<br><br>*Probe: Did you have enough information about what was going to happen during the admission process?*
	**Experience during hospitalization**	
Experiences of the Integrated Care program: Admission to hospital process<br><br>Perceived efficacy: Person-centred care<br><br>Perceived efficacy: Interactions, communications, and information continuity<br><br>Perceived efficacy: Coordination and timeliness of care	**Question 2:** Can you tell me about your experiences with the care you received from nurses, doctors, and other health care professionals while you were in the hospital?<br><br>*Probes:*<br>- *Did nurses, doctors, and health care professionals listen carefully to you?*<br>- *Did nurses, doctors, and health care professionals explain things in a way you could understand?*<br>- *Was your admission into the hospital well-organized?*<br>- *Were hospital staff prepared for your admission?*	**Question 2.** Can you tell me about your experiences with the nurses, doctors, and other health care professionals while the patient stayed in the hospital?<br><br>*Probes:*<br>- *Did nurses, doctors, and health care professionals listen to your concerns about the patient?*<br>- *Did nurses, doctors, and health care professionals explain things in a way you could understand?*<br>- *Was the patient's admission into the hospital well-organized?*<br>- *Were hospital staff prepared for the patient's admission?*

Experiences of the Integrated Care program: Internal coordination of care<br><br>Perceived efficacy: Interactions, communications, and information continuity	**Question 3:** Can you tell me about your experiences with the communication about your care between doctors, nurses, and other hospital staff?<br><br>*Probe: Did you feel that doctors, nurses, and other hospital staff seemed informed and up to date about your hospital care?*	**Question 3.** Can you tell me about your experiences with the communication about the patients care between doctors, nurses, and other hospital staff?<br><br>*Probe: Did you feel that doctors, nurses, and other hospital staff seemed informed and up to date about the patient's hospital care?*
Perceived efficacy: Education and Knowledge	**Question 4:** During your hospital stay, did you receive all the information you needed about your condition, treatment, and medications?	**Question 4.** During the hospital stay, did you receive all the information you needed about the patient's condition, treatment, and medications?
Perceived efficacy: Person-centred care<br><br>Perceived efficacy: Support for patient preferences and family involvement	**Question 5:** During your hospital stay, were you involved as much as you wanted to be in decisions about your care and treatment?<br><br>*Probe: Were your family and friends involved as much as you wanted in decisions about your care and treatment in the hospital?*	**Question 5.** During the hospital stay, were you involved as much as you wanted to be in decisions about the patients care and treatment?
Experiences of the Integrated Care program: Global hospital experience<br><br>Perceived efficacy: Overall satisfaction	**Question 6:** Overall, how do you feel about your experience during your hospital stay?	**Question 6.** Overall, how do you feel about your experience during the patients stay in the hospital?
**Experience with transition from hospital to home and post-discharge care**		
Experiences of the Integrated Care program: Discharge, transition, & post-discharge process<br><br>Experiences of the Integrated	**Question 7**: Were you involved as much as you wanted to be in the planning for your discharge from the hospital?<br><br>*Probe: Did hospital staff take your preferences and those of your family or caregiver into account in deciding what your health care needs would be when you left the hospital?*	**Question 7**: Were you involved as much as you wanted to be in the planning for the patients discharge from the hospital?

Care program: Involvement in transition planning<br><br>Perceived efficacy: Support for patient preferences		
Experiences of the Integrated Care program: Discharge, transition & post-discharge process<br><br>Perceived efficacy: Education and knowledge<br><br>Perceived efficacy: Interactions and communications<br><br>Outcome: Perceived caregiver preparedness	**Question 8:** Before leaving the hospital and returning home, did you have the information and support you needed to manage your health after your hospital stay?<br><br>*Probes:*<br>- *Did you have enough information about your dietary needs?*<br>- *Did you have a good understanding of the things you were responsible for in managing your health?*<br>- *Did you have enough information from hospital staff about what to do if you had questions or were worried about your condition or treatment?*<br>- *Did doctors, nurses or other hospital staff talk with you about whether you would have the help you needed when you left the hospital?*	**Question 8.** When you left the hospital, how well prepared did you feel for your caregiving role?<br><br>*Probes:*<br>- *Were there aspects of care giving that you felt well prepared for?*<br>- *Were there aspects of care giving that you felt unprepared for?*<br>- *Did you have enough information about the patient's dietary needs?*<br>- *Were you given enough information on how to provide care or help the patient at home?*<br>- *Did you have enough information from hospital staff about what to do if you had questions or were worried about the patient's condition or treatment?*<br>- *Were you given enough information about the patient's condition, treatment, and prescribed medications?*<br>- *Did anybody from the hospital staff ask you if you were able or willing to help with the patients care?*<br>- *Did you have enough information on available caregiver support services (e.g., caregiver support groups, educational seminars) or on how to care for yourself?*<br>- *Did you have enough information about what*

<table>
<tr><td></td><td></td><td>publicly funded services were available to the patient and what services you would need to pay for?</td></tr>
<tr><td>Experiences of the Integrated Care program: Continuity of care

Perceived efficacy: Interactions and communications</td><td>Question 9: After you left the hospital, did the doctors or staff at the place where you usually get medical care (e.g., family doctor) seem informed and up to date about the care you received in the hospital?

Probe: If you received home care, did the home care team seem informed and up to date about the care you received in hospital? By home care team, we mean the care providers you saw after being discharged from UHN.</td><td>Question 9. After the patient was discharged, did the doctors or staff at the place where the patient usually gets medical care seem informed and up to date about the care the patient received in the hospital? (e.g., the patients family doctor)

Probe: If the patient received home care, did the home care team seem informed and up to date about the care the patient received in hospital? (By home care team, we mean the care providers you saw after being discharged from University Health Network)</td></tr>
<tr><td>Integrated care resource utilized: Perceived efficacy

Experiences of the Integrated Care program: Discharge, transition, & post-discharge process

Perceived efficacy: Coordination and timeliness of care

Perceived efficacy: Access to care & convenience</td><td>Question 10a: Just for my understanding, can you tell me what integrated care services were offered to you after you left the hospital and returned home?

Probe: Did you receive home care services, telephone support, access to an integrated care lead, services from any professionals, such as a dietician or speech language pathologist?

Question 10b: Can you tell me about your experiences with these services that you received after leaving the hospital and returning home?

Probes:
- How useful were the resources that were offered through the Integrated Care program?
- Were members of the home care team available when you needed them?
- Were your home care visits arranged in a manner that was convenient for you?
- Do you feel that you received the right amount of home care and services you needed when you returned home?</td><td>Question 10a. Just for my understanding, can you tell me what types of integrated care services that were offered to the patient you cared for after they left the hospital and returned home?

Probe: Did they receive home care services, telephone support, access to an integrated care lead, or services from any professionals, such as a dietician or speech language pathologist?

Question 10b. Can you tell me about your experiences with these services that the patient received after leaving the hospital and returning home?

Probes:
- How useful were the resources that were offered through the Integrated Care program?
- Were members of the home care team available when you needed them?
- Were home care visits arranged in a manner that was convenient for you?</td></tr>
</table>

	- *Were there times that you felt abandoned by the health care system or didn't know where to turn for help?*	- *Do you feel that you received the right amount of home care and services that you needed when you returned home?*<br>- *Were there times when you were confused about the role of different service providers?*<br>- *Were there times that you felt abandoned by the health care system or didn't know where to turn for help?*<br>- *Did you or the patient experience any issues or challenges that impacted the care that the patient received at home?*
Experiences of the Integrated Care program: Continuity of care<br><br>Outcome: Emergency department visits / readmission to hospital	**Question 11:** After your left the hospital, did you have to visit the emergency department or be readmitted to the hospital for any reason?<br><br>**If yes,**<br>- Do you feel that this visit could have been prevented? How?<br>- Did you try to get help elsewhere before visiting the emergency department, such as from your family doctor?<br>**If no,**<br>Did you feel that the care you received helped you to avoid a visit to the emergency department or hospital? How?	**Question 11.** After the patient left the hospital, did the patient have to visit the emergency department or be readmitted to the hospital for any reason?<br>**If yes,**<br>- Do you feel that this visit could have been prevented? How?<br>**If no,**<br>- Do you feel that the care the patient received helped the patient to avoid a visit to the emergency department or hospital? How?
Perceived efficacy: Overall satisfaction<br><br>Perceived efficacy: Person-centred care	**Question 12:** Overall, do you feel you were supported after you left the hospital and returned home?<br><br>*Probe: Did you experience any issues or challenges that impacted the care you received at home?*	
**Patient and caregiver reported health outcomes**		
Outcome: Perceived patient confidence to self-manage their health<br><br>Outcome: Patient	**Question 13:** How did the program impact your ability to manage your health?<br><br>*Probe: Did the program help you feel confident about your ability to take care of your health?*	**Question 12.** How do you feel your health was impacted by your caregiving responsibilities while the patient was in the Integrated Care program?<br><br>*Probes:*<br>- *Were the times that you felt you did not have enough time for*

preparedness<br><br>Outcome: perceived caregiver wellbeing & burden		*yourself because of the time you spend with the patient?*<br>- *Were there times that you felt stressed between caring for the patient and trying to meet other responsibilities for your family and work?*<br>- *Were there times that you felt strained when you were around the patient?*<br>- *How did the Integrated Care program help you cope with your caregiving responsibilities?*
Outcome: Perceived patient physical & emotional health	**Question 14:** How did the program impact your health, wellbeing, or quality of life?	
	**Question 15:** Is there anything else that you would like to share about your experiences in the Integrated Care program that we haven't discussed today?	**Question 13:** Is there anything else that you would like to share about your experiences in the Integrated Care program that we haven't discussed today?
