# Supplementary material for: Experiences and Reported Outcomes of Patients and Caregivers Enrolled in an Integrated Care Program for Thoracic Surgery: A Qualitative Evaluation
Source: Int J Integr Care. 2023 May 4;23(2):11. doi: 10.5334/ijic.6540 (PMC10162348; doi:10.5334/ijic.6540)
Supplement: S2 Table. — Qualitative analysis coding manual. [file ijic-23-2-6540-s2.pdf]

S2 Table. Qualitative Analysis Coding Manual

Code	Sub code	Description
**Context / situation**		• General information about the hospital, department, program, providers, patients, caregivers, etc. that will allow us to situate the evaluation in a larger context
**Integrated Care Experience – pre-hospital**	Pre-hospital admission process	• Mention or description (positive, negative, neutral) of experiences prior to when the patient was admitted into the hospital for their surgery. Examples include receiving resources prior to the hospital admission.
**Integrated Care Experience – hospitalization**	Admissions to hospital - inpatient	• Mention or description (positive, negative, neutral) of experiences when the patient was admitted into the hospital for their surgery. Examples include information-sharing with health care providers on admission.
	Internal coordination of care & communications - inpatient	• Mention or description (positive, negative, neutral) of experiences with the coordination and communication of patients care during the hospitalization.
	Person-centred care - inpatient	• Mention or description (positive, negative, neutral) of experiences with patient or caregiver involvement with decisions about their care during the hospitalization. Person centred care has been defined as care that is focused and organized around the health needs and expectations of patients, rather than communities.
	Global Hospital Experience	• Mention or description (positive, negative, neutral) of the overall experience of the hospital stay
**Integrated Care Experience – post-discharge**	Discharge, transition and post-discharge process	• Mention or description (positive, negative, neutral) of experiences with the discharge, transition process or post-discharge experience. Examples include coordination and communication of patients transition planning, arrangement of home care, transfer of transition plans, and information related to patients care.
	Involvement in transition planning	• Mention or description (positive, negative, neutral) of patient/caregiver involvement in decisions about transition planning
	Continuity of care	• Mention or description (positive, negative, neutral) of experiencing care over time as coherent and linked. Examples include communicating patient needs to appropriate health care providers, coordinating care. Continuity of care has been defined as the degree to which a series of discrete health care events are experiences by people as coherent and interconnected over time and are consistent with their health needs and preferences.
**Integrated Care – Efficacy**	Efficacy of integrated care resources	• Mention or description (positive, negative, neutral) of efficacy of integrated care resources provided or utilized

Code	Sub code	Description
		(e.g., home care visit, phone call, allied services).
	Education and knowledge	• Mention or description of efficacy of supports offered to patients/caregivers for their education and knowledge. Mention or description (positive, negative, neutral) of patient/caregiver having information and support they need to manage their health after the hospital stay, offering of education and training to manage health care needs at home, guidance on medications or medical equipment, and/or preparation for caregiving.
	Interactions, communications, and informational continuity	• Mention or description of efficacy of interactions and communications between health care providers or between patients/caregivers and health care providers to manage patients care. Examples include health care providers being informed and up to date about patient care. Informational continuity helps ensure that when many different providers are involved in caring for individuals, communication of information is seamless, up to date, efficient and accurate. Mention or description of efficacy of information flow between health care providers after patient was discharged from the hospital. Examples include providers being aware of patient issues.
	Coordination & timeliness of care	• Mention or description of efficacy of coordination of patients care and timeliness of care. Examples include coordination of home care, follow-up care.
	Access to care and convenience	• Mention or description of efficacy of access to care and convenience. Examples include responding to patient concerns. Mention or description (positive, negative, neutral) of experience with access to care post-discharge. Examples include addressing patient concerns, arranging follow-up care, home care services
	Support for patient preferences and family involvement	• Mention or description of efficacy of involving patients and caregivers in decisions about their care. This concept can be considered a form of person-centred care.
	Overall satisfaction	• Mention or description of efficacy or satisfaction with care received or provided through the integrated care program overall (pre-admission, during hospitalization, post-discharge).
	Recommendations	• Mention of recommendations for the program (e.g., recommendations for improvement around dimensions of care, integrated care resources, sustainability, etc.).
**Patient/ Caregiver Reported Outcomes**	Perceived patient confidence to self-manage their health	• Mention or description (positive, negative, neutral) of patient's level of confidence or preparedness to manage their health after their hospital stay
	Perceived caregiver	• Mention or description (positive, negative, neutral) of

Code	Sub code	Description
	or patient preparedness	caregiver’s preparedness for caregiving role. Examples include level of knowledge and skills to provide care or access to supports. Mention or description (positive, negative, neutral) of patient feeling prepared for the surgery, hospital admission, post-discharge, or other aspects of treatment and recovery
	Perceived patient physical or emotional health	• Mention or description (positive, negative, neutral) of patients reported health. Examples include mental and physical wellbeing.
	Perceived caregiver wellbeing / burden	• Mention or description (positive, negative, neutral) of caregivers reported wellbeing or caregiving burden.
	ED visit or readmission	• Mention or description of experiences with an emergency department visit or hospital readmissions. Examples include avoiding a emergency department visit or readmission or experiences of being admitted to the emergency department or hospital readmission
